# Draft genome sequence dataset and genomic features of *Oceanimonas baumannii* strain ICA-29, a shrimp-associated bacterium isolated from a commercial aquaculture system

**DOI:** 10.1016/j.dib.2026.113280

**Published:** 2026-09-24

**Authors:** Guillermo Reyes, Martha Borbor, Ramiro Solórzano, Juan Carlos Muñoz, Rosa Malavé, Betsy Andrade, Bonny Bayot

**Affiliations:** a Centro Nacional de Acuicultura e Investigaciones Marinas, CENAIM, Escuela Superior Politécnica del Litoral, ESPOL, Guayaquil, Ecuador; b Facultad de Ingeniería Marítima y Ciencias del Mar, FIMCM, Escuela Superior Politécnica del Litoral ESPOL, Guayaquil, Ecuador

**Keywords:** *Penaeus vannamei*, Aquaculture, AHPND, *Vibrio parahaemolyticus*, Whole-genome sequencing, Phylogenomics, Comparative genomics

## Abstract

Bacterial diseases represent a major challenge for *Penaeus vannamei* shrimp aquaculture. Here, we report the draft genome sequence and genomic characterization of *Oceanimonas baumannii* strain ICA-29, isolated from juvenile shrimp collected in a commercial pond affected by acute hepatopancreatic necrosis disease (AHPND). Illumina sequencing yielded a 3.58 Mb draft genome assembled into 42 contigs, with a 55.94% G+C content and 99.21% completeness. Taxonomic assignment was supported by phylogenomic analysis, an average nucleotide identity (ANI) value of 99.22%, and a digital DNA-DNA hybridization (dDDH) value of 93.50% relative to the reference *O. baumannii* strain DSM 15594. Genome annotation identified 3,360 coding sequences (CDSs). COG functional profiling revealed a predominance of metabolism-related functions, followed by cellular processes and signaling, and information storage and processing. Screening against antimicrobial resistance and virulence factor databases identified only low-confidence homology matches to antimicrobial resistance-associated entries and several putative homologs to entries contained in the Virulence Factor Database (VFDB), primarily related to conserved polar and lateral flagellar motility functions. These data provide a genomic resource for comparative analyses of *Oceanimonas* species and for the investigation of bacterial traits relevant to aquaculture systems.

Specifications Table

**Table:** 

Subject	Biology
Specific subject area	Bacterial genomics*.*
Type of data	Tables, Figures Raw, Analyzed, Processed and Deposited.
Data collection	A bacterial strain (ICA-29) was isolated from *P. vannamei* shrimp collected from a commercial pond affected by AHPND. The strain was selected for genome sequencing following preliminary observations obtained during strain screening. Genomic DNA: Quick-DNA HMW MagBead Kit; DNA quality assessment: DS-11 spectrophotometer and 2% agarose gel electrophoresis; Sequencing: Illumina NovaSeq 6000; Assembly: Shovill; Annotation: PGAP; Taxonomic assignment and phylogenomics: TYGS; Average nucleotide identity (ANI): FastANI; digital DNA-DNA hybridization (dDDH): Genome-to-Genome Distance Calculator (GGDC). Functional annotation: eggNOG; Virulence factor screening: VFDB; Antimicrobial resistance gene screening: CARD, ARG-ANNOT, ResFinder, and MEGARes.
Data source location	The bacterium was isolated from *P. vannamei* shrimp collected from a commercial pond in South America.
Data accessibility	All raw sequencing data and genome assemblies have been deposited in publicly accessible repositories prior to submission. Repository name: National Center for Biotechnology Information (NCBI) The deposited records include raw Illumina sequencing reads, genome assembly, and associated sample metadata. Data identification numbers: BioProject: PRJNA1478750 BioSample: SAMN60909231 Genome assembly (GenBank): JBZVGB000000000 Direct URLs to data Sequence Read Archive (SRA) and Genbank: https://www.ncbi.nlm.nih.gov/bioproject/PRJNA1478750/. https://www.ncbi.nlm.nih.gov/nuccore/JBZVGB000000000/. The repository provides unrestricted public access to all data generated and analyzed.
Related research article	None*.*

## 1 Value of the Data

• These data provide a genome sequence resource for an *Oceanimonas baumannii* strain isolated from *Penaeus vannamei* shrimp, thereby expanding the genomic information available for this species in aquaculture environments.
• The dataset provides reference genomic resources for studies of *Oceanimonas* diversity and taxonomy.
• These data can be reused in comparative genomic analyses of bacteria isolated from aquaculture systems, as well as for identifying genomic traits of interest for aquaculture applications.

## 2 Background

*Penaeus vannamei* is the most widely cultured shrimp species worldwide, and bacterial diseases remain a significant challenge in commercial shrimp production [1,2]. Interest in characterizing microorganisms from aquaculture environments has increased as part of efforts to identify bacteria associated with shrimp health [3]. Strain ICA-29 was isolated from *P. vannamei* juvenile shrimp cultured in a commercial shrimp pond affected by acute hepatopancreatic necrosis disease (AHPND), which is mainly caused by *Vibrio parahaemolyticus* [4]. Although AHPND was reported during the production cycle, the pond achieved an 85% survival rate at harvest. Preliminary observations during the present project suggested potential antagonistic activity by strain ICA-29 against a pathogenic *V. parahaemolyticus* strain that causes AHPND in shrimp. These observations are not included in the dataset reported and served only as background information contributing to the selection of ICA-29 for whole-genome sequencing. The dataset includes the genomic characterization of strain ICA-29, identified as *Oceanimonas baumannii* through whole-genome analyses, including raw sequencing reads, genome assembly, and annotation.

## 3 Data Description

This article presents the draft genome sequence and genomic features of *O. baumannii* strain ICA-29, isolated from juvenile *P. vannamei* shrimp. The dataset includes project (BioProject PRJNA1478750) and sample (BioSample SAMN60909231) metadata, raw Illumina sequencing reads (SRA SRR39207378), a draft genome assembly (GenBank JBZVGB000000000), and the corresponding genome annotation files for *O. baumannii* strain ICA-29. The draft genome was assembled into 42 contigs totaling 3,581,163 bp (3.58 Mb), with a G+C content of 55.94% (Table 1). The largest contig was 413,626 bp, while the assembly showed an N50 of 183,605 bp and an L50 of 7 contigs (Table 1). Genome completeness was estimated at 99.21%, with 0.70% contamination (Table 1). A total of 35 contigs were assigned to the *Oceanimonas* genus after taxonomic validation by BLASTn analysis, whereas the remaining seven contigs corresponded only to short no-hit sequences (Fig. 1). Detailed contig-level metrics, including G+C content, contig length, mean read depth, and taxonomic assignment for the 42 contigs, are provided in Supplementary Table 1. Whole-genome-based phylogenomic analysis clustered strain ICA-29 with the reference strain *O. baumannii* DSM 15594 (GenBank accession GCA_004365405) (Fig. 2). Comparative genome analyses showed an ANI value of 99.22%, a dDDH value of 93.5% (GGDC Formula 2; 95% CI: 91.7-95.0%), and a G+C content difference of 0.03% relative to *O. baumannii* DSM 15594 (Supplementary Table 2). TYGS assigned strain ICA-29 and *O. baumannii* DSM 15594 to the same species cluster (cluster 6) and subspecies cluster (cluster 12), further supporting the classification of strain ICA-29 as *O. baumannii* (Supplementary Table 3). Genome annotation identified 3,438 genes and 18 pseudogenes, including 3,360 predicted coding sequences (CDSs), six rRNA genes, 68 tRNA genes, and four ncRNA genes (Table 1, Fig. 3). The distribution of Clusters of Orthologous Groups (COGs) functional assignments among predicted coding sequences (CDSs) is shown in Fig. 4. COG functional classification was dominated by metabolism-related functions (38.7%), followed by cellular processes and signaling (31.6%), and information storage and processing (18.4%), with 11.3% corresponding to poorly characterized functions (Fig. 4). Regarding functional annotation, the COG categories with the highest number of functional assignments included amino acid transport and metabolism (336), translation, ribosomal structure and biogenesis (275), transcription (273), general function prediction only (270), cell wall/membrane/envelope biogenesis (262), signal transduction mechanisms (244), energy production and conversion (237), and inorganic ion transport and metabolism (218). *In silico* screening against the antimicrobial resistance gene databases: CARD, ARG-ANNOT, ResFinder, and MEGARes identified two low-confidence homology matches. The first match corresponded to the *vatF* gene, identified in contig Oceanimonas_bactopia_00001 through the CARD database (63.51% coverage, 71.43% identity), which is annotated as a streptogramin A acetyltransferase. However, this level of partial similarity was insufficient to establish the presence of a functional streptogramin A acetyltransferase. The second match corresponded to the *CRP* gene in contig Oceanimonas_bactopia_00012 through the CARD database (99.05% coverage; 77.39% identity). Because CRP is a broadly distributed global transcriptional regulator, this match was not interpreted as evidence of an antimicrobial resistance determinant in strain ICA-29. Consequently, no high-confidence antimicrobial resistance determinants were identified in the assembled genome. Screening against the VFDB database identified 33 putative homologs to VFDB entries, most of which correspond to common bacterial functions, including flagellar assembly, chemotaxis, *rpoS, fur, luxS, tufA, htpB*, and *katB* (Table 2). However, these matches do not demonstrate that the corresponding genes function as virulence determinants in *O. baumannii* ICA-29 and should be interpreted only as sequence homology-based annotations, particularly because many exhibited only 70-80% identity to genes from unrelated pathogenic reference organisms (Table 2). Similarly, the detection of *LuxS* does not indicate AI-2-mediated quorum sensing or a biofilm-associated phenotype because LuxS also participates in central bacterial metabolism.

**Table 1:** Assembly details and annotated genome features of the *Oceanimonas baumannii* strain ICA-29.

Features	Values
Genome assembly	
Genome size (bp)	3,581,163
G+C content (%)	55.94
Number of contigs	42
Largest contig (bp)	413,626
Contig N50 (bp)	183,605
Contig L50	7
CheckM2 completeness (%)	99.21
CheckM2 contamination (%)	0.70
Genomic annotation	
Total genes	3,438
Protein-coding genes (CDSs)	3,360
Pseudogenes	18
rRNA genes	6 (5S = 2, 16S = 1, 23S = 3)
tRNA genes	68
ncRNA genes	4
CRISPR arrays	1

**Fig. 1:**
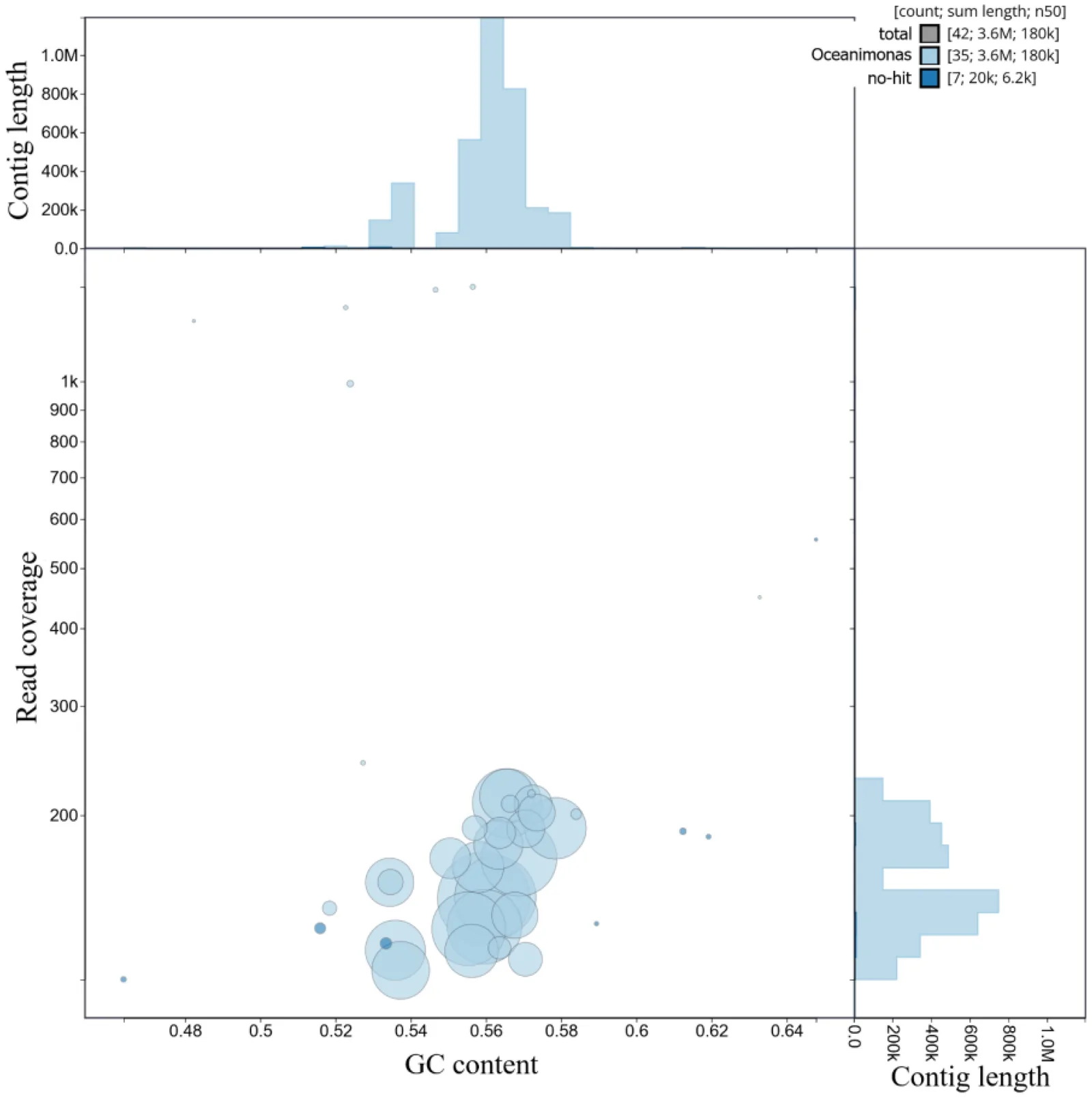
BlobToolKit analysis of the draft genome of *Oceanimonas baumannii* strain ICA-29. The plot shows the distribution of assembled contigs according to G+C content and sequencing coverage. Most contigs were taxonomically assigned to the genus *Oceanimonas*, while a smaller number showed no significant taxonomic match (no-hit). Bubble size represents contig length, and the marginal histograms show the distribution of contig lengths across the two plotted variables.

**Fig. 2:**
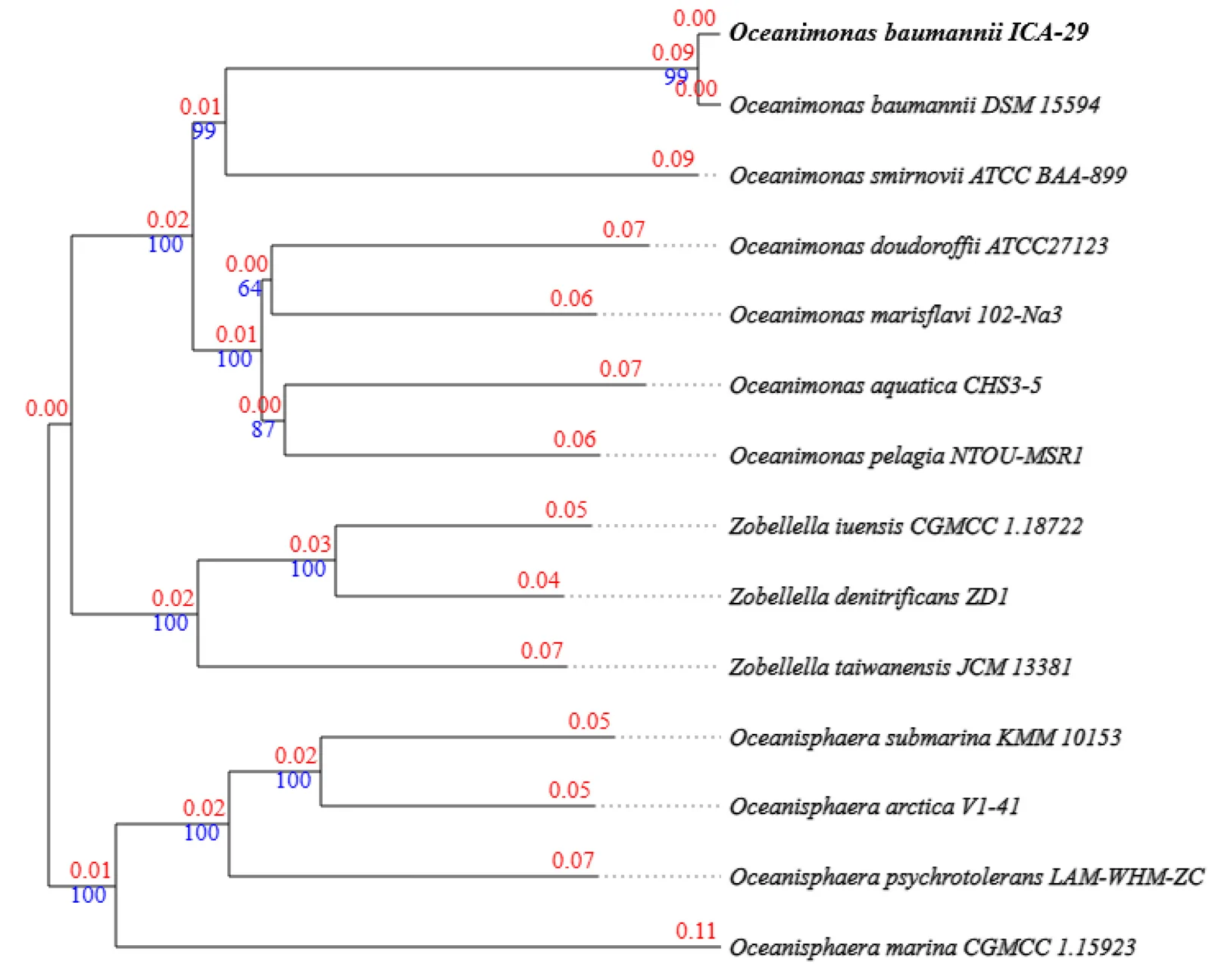
Genome-based phylogenomic tree showing the position of strain ICA-29 among type strains of the genus *Oceanimonas* and related taxa. Strain ICA-29 clustered with *Oceanimonas baumannii* DSM 15594 (GenBank accession GCA_004365405). Blue numbers at branching points indicate pseudo-bootstrap support values (%). Branch lengths (red numbers) represent genomic distances inferred by the Type (Strain) Genome Server (TYGS).

**Fig. 3:**
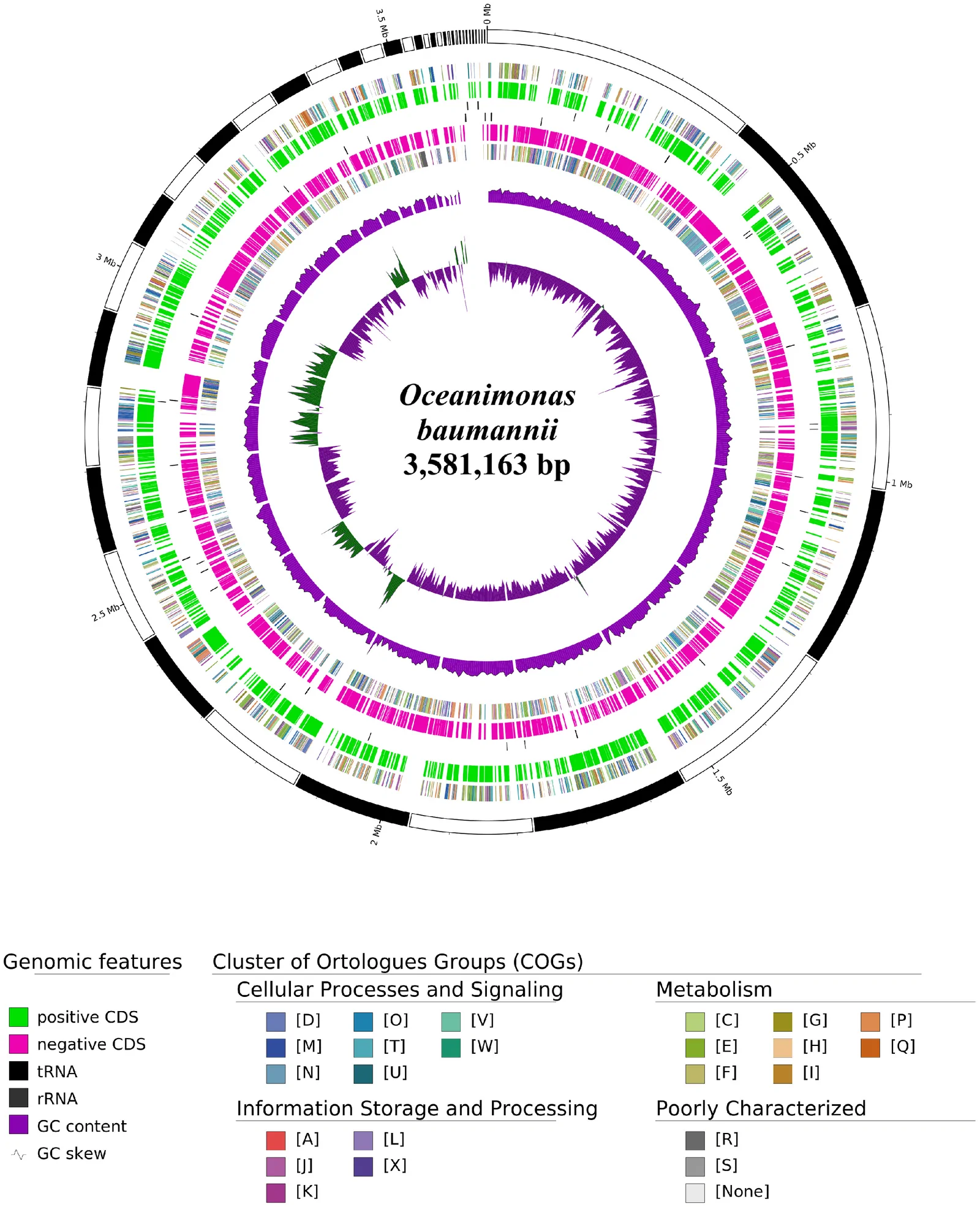
Circular visualization of the draft genome of *Oceanimonas baumannii* strain ICA-29. Circles from the outside to inside show coding sequences (CDSs) on the positive strand, CDSs on the negative strand, tRNA genes, rRNA genes, COG functional categories, G+C content, and G+C skew. Functional annotation was based on the Clusters of Orthologous Groups (COG) database. The color key indicates the corresponding COG categories and genomic features. The outermost ring represents the 42 contigs, with gaps between adjacent segments indicating contig boundaries.

**Fig. 4:**
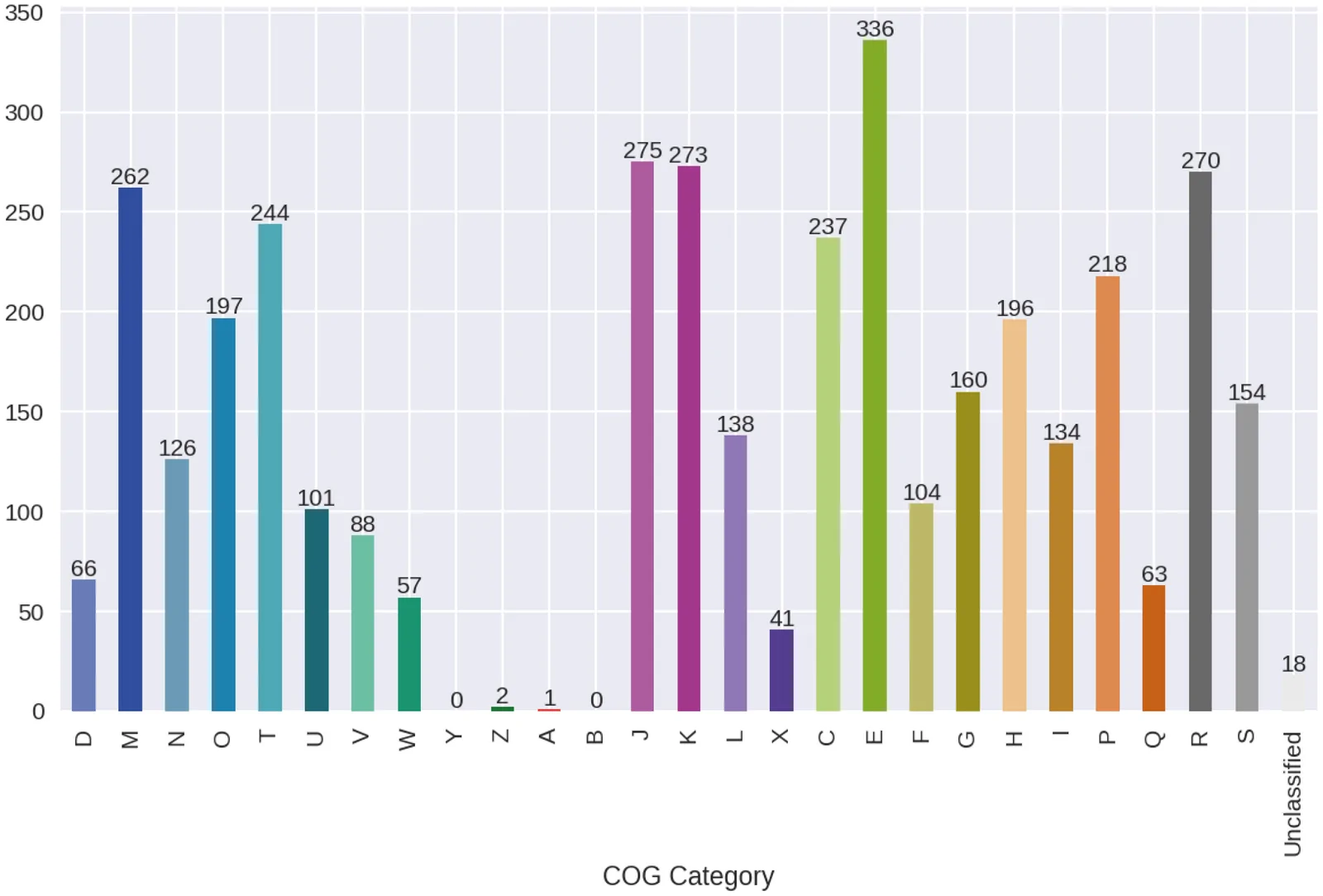
Distribution of predicted coding sequences (CDSs) among Clusters of Orthologous Groups (COG) functional categories in *Oceanimonas baumannii* strain ICA-29. Bars represent the number of COG assignments in each functional category.

**Table 2:** Putative homologs to Virulence Factor Database (VFDB) entries identified in the draft genome of *Oceanimonas baumannii* strain ICA-29, categorized by functional profile and reference homologs.

Gene (VFDB ID)	Coverage (%)	Identity (%)	VFDB Functional Category	Reference Organism
*gmhA/lpcA (VFG013418)*	72.82	72.07	Immune modulation / Lipooligosaccharide (LOS)	*Haemophilus influenzae* Rd KW20
*pomA2 (VFG038836)*	87.09	72.10	Motility / Polar flagella	*Aeromonas hydrophila* ML09-119
PA1464 (VFG043016)	90.42	76.04	Motility / Flagella	*Pseudomonas aeruginosa* PAO1
*cheB-2* (VFG038711)	93.73	71.98	Motility / Polar flagella	*Aeromonas hydrophila* ML09-119
*cheY* (VFG038699)	100.00	78.39	Motility / Polar flagella	*Aeromonas hydrophila* ML09-119
*fliA* (VFG038695)	86.94	70.08	Motility / Polar flagella	*Aeromonas hydrophila* ML09-119
*flhG* (VFG038691)	71.80	73.77	Motility / Polar flagella	*Aeromonas hydrophila* ML09-119
*flhF* (VFG038687)	61.11	71.88	Motility / Polar flagella	*Aeromonas hydrophila* ML09-119
*flhA* (VFG038683)	96.43	73.65	Motility / Polar flagella	*Aeromonas hydrophila* ML09-119
*fliQ* (VFG038671)	77.41	75.12	Motility / Polar flagella	*Aeromonas hydrophila* ML09-119
*fliP* (VFG038667)	88.33	78.95	Motility / Polar flagella	*Aeromonas hydrophila* ML09-119
*fliN* (VFG038659)	67.94	83.89	Motility / Polar flagella	*Aeromonas hydrophila* ML09-119
*fliM* (VFG038655)	96.27	74.86	Motility / Polar flagella	*Aeromonas hydrophila* ML09-119
*fliI* (VFG038639)	93.77	72.32	Motility / Polar flagella	*Aeromonas hydrophila* ML09-119
*fliG* (VFG038631)	92.47	74.95	Motility / Polar flagella	*Aeromonas hydrophila* ML09-119
*flgI* (VFG038780)	94.35	74.33	Motility / Polar flagella	*Aeromonas hydrophila* ML09-119
*flgG* (VFG038788)	98.99	73.88	Motility / Polar flagella	*Aeromonas hydrophila* ML09-119
*flgF* (VFG038792)	99.60	70.00	Motility / Polar flagella	*Aeromonas hydrophila* ML09-119
*flgC* (VFG038804)	95.24	72.50	Motility / Polar flagella	*Aeromonas hydrophila* ML09-119
*lfgG* (VFG038876)	99.87	73.19	Motility / Lateral flagella	*Aeromonas salmonicida* A449
*fliP* (VFG038857)	76.87	72.60	Motility / Lateral flagella	*Aeromonas salmonicida* A449
*rpoS* (VFG000477)	81.67	74.23	Regulation / RpoS	*Salmonella enterica* LT2
*tapU* (VFG038468)	77.84	72.82	Adherence / Tap type IV pili	*Aeromonas hydrophila* ML09-119
*tapT* (VFG038464)	92.95	78.36	Adherence / Tap type IV pili	*Aeromonas hydrophila* ML09-119
*tufA* (VFG046465)	99.75	71.42	Adherence / EF-Tu	*Francisella tularensis* SCHU S4
*flmH* (VFG038840)	99.59	77.66	Motility / Polar flagella	*Aeromonas hydrophila* ML09-119
*fur* (VFG000478)	87.64	74.31	Regulation / Fur	*Salmonella enterica* LT2
*htpB* (VFG001855)	98.55	70.94	Adherence / Hsp60	*Legionella pneumophila* Phil. 1
*luxS* (VFG018241)	82.27	73.19	Biofilm / AI-2	*Vibrio cholerae* O1 El Tor N16961
*katB* (VFG001862)	95.11	70.94	Stress survival / KatAB	*Legionella pneumophila* Phil. 1

## 4 Experimental Design, Materials, and Methods

Strain ICA-29 was obtained from a pooled intestinal macerate of ten juvenile shrimp cultured in a commercial shrimp pond. Because the intestinal material originated from a pooled sample, the isolate could not be assigned to a specific host individual. The sample was preserved in PBS supplemented with 20% glycerol and stored at −80°C. For bacterial recovery, 20 µL of the preserved sample was inoculated into 200 µL of saline solution, homogenized, and 20 µL of the suspension was transferred into tubes containing 5 mL of Tryptic Soy Broth (TSB). Cultures were incubated at 30°C for 48 h. After turbidity was observed, aliquots were streaked onto Tryptic Soy Agar (TSA) supplemented with 2% NaCl, and the plates were incubated at 30°C for 24 h. Two predominant colony morphotypes were recovered and morphologically characterized. Colonies corresponding to isolate ICA-29 were medium-sized, irregular, cream-colored, and rough, whereas the second predominant morphotype consisted of large, irregular, cream-colored colonies. Gram staining was performed according to Beveridge (2001) [5], and both isolates were characterized as Gram-negative. Individual colonies of each morphotype were isolated by streak plating on TSA supplemented with 2% NaCl and incubated at 30°C for 24 h. Purity of isolate ICA-29 was evaluated based on the recovery of isolated colonies displaying a single and consistent colony morphology. A single colony of isolate ICA-29 was then inoculated into TSB supplemented with 2% NaCl, mixed with sterile glycerol to a final concentration of 20%, and stored at −80°C for subsequent analyses. Preliminary observations of potential antagonistic activity against an AHPND-associated *V. parahaemolyticus* isolate, which are not part of the dataset reported in this article, contributed to the selection of strain ICA-29 for genome sequencing.

High-molecular-weight (HMW) DNA was isolated utilizing the Quick-DNA HMW MagBead Kit (Zymo Research, USA). The integrity and concentration of the extracted DNA were subsequently evaluated via agarose gel electrophoresis and a DeNovix DS-11 spectrophotometer (DeNovix Inc., USA). Finally, library preparation and whole-genome sequencing were outsourced to Novogene Inc. (Sacramento, USA), employing the Illumina NovaSeq 6000 platform to generate 2 × 150 bp paired-end reads. A total of 23.67 million raw reads (∼3.57 Gb) were obtained, with a Q30 quality score of 92.37% and a duplication rate of 4.23%. Quality filtering using fastp retained approximately 23.38 million quality-filtered reads (3.53 Gb; 98.78% retention rate). The draft genome assembly was performed using Bactopia v4.0.0, which uses Shovill v1.1.0 with SKESA v2.5.1 as the assembly engine [6]. Prior to assembly, the quality-filtered reads were subsampled by the Bactopia workflow using BBMap ReformatReads to approximately 600 Mb (3,976,284 reads; 17.01% of the filtered dataset). Based on this processed dataset, Shovill estimated a sequencing depth of approximately 200× and did not perform additional read-depth reduction. Overlapping paired-end reads were merged using FLASH, and final polishing was performed using BWA, SAMtools, SAMclip, and Pilon. Genome quality (completeness and contamination) of *Oceanimonas* strain ICA-29 was evaluated using CheckM2 v1.1.0 [7]. The subsampled read set used for assembly was mapped back to the final assembly using Bowtie2 and SAMtools, yielding a 99.61% read-mapping rate. Each contig was individually evaluated based on read depth (coverage), G+C content, and taxonomic assignment using BlobToolKit [8], with taxonomic identities further assessed using NCBI BLASTn. Genome-wide read depth was independently estimated by mapping the original quality-filtered reads against the final 3,581,163-bp assembly using BWA-MEM, processing alignments with SAMtools, and calculating depth with Mosdepth, yielding a mean mapped read depth of 943.04×. The assembled FASTA file was uploaded to the NCBI Prokaryotic Genome Annotation Pipeline (PGAP, v2026) [9] for annotation and CRISPR identification. The resulting GenBank (.gbk) file was used to generate the circular visualization of the draft assembly using GenoVi and to perform functional annotation based on COGs using eggNOG [10].

Taxonomic delimitation was established by calculating Average Nucleotide Identity (ANI) values using fastANI within the Proksee platform [11]. In addition, dDDH values (Formula 2; d4) were estimated using the Genome-to-Genome Distance Calculator (GGDC 3.0; DSMZ), with the BLAST+ alignment tool. Species boundaries were evaluated using the generally accepted thresholds of 95–96% for ANI and 70% for dDDH. Genomic data were screened against virulence factor and antimicrobial resistance databases using ABRicate v1.4.0. Putative homologs of entries contained in the Virulence Factor Database (VFDB, set A-core dataset) were identified based on sequence similarity [12]. Putative antimicrobial resistance-associated matches were identified by screening against the CARD [13], ARG-ANNOT, ResFinder, and MEGARes databases. For all screenings, putative homology matches were retained only if they met a minimum threshold of 70% sequence identity and 60% alignment coverage.

To resolve the phylogenetic positioning of *Oceanimonas* strain ICA-29, a comprehensive phylogenomic reconstruction was executed via the Type Strain Genome Server (TYGS) pipeline [14,15], adhering to the current taxonomic nomenclature curated by the LPSN database [15]. The comparative workflow initiated with a MASH-based min-hash screening of the ICA-29 assembly against the global TYGS type-strain repository, enabling the selection of the closest genomic neighbors. Evolutionary distances were subsequently calculated using the Genome BLAST Distance Phylogeny (GBDP) approach governed by the distance formula *d5*. Nodal stability on the resulting topology was rigorously evaluated through 100 pseudo-bootstrap replicates, with branch lengths scaled proportionally to GBDP distances. Tree rendering and visualization were completed directly within the TYGS framework. TYGS outputs were further used to determine species and subspecies cluster assignments for taxonomic validation.

## Limitations

This dataset corresponds to a draft genome assembly of *O. baumannii* strain ICA29 generated from Illumina short-read sequencing data. Consequently, the genome is not completely closed, and some repetitive or structurally complex regions may remain unresolved.

## Ethics Statement

Juvenile *P. vannamei* shrimp were collected from a commercial production pond as part of routine production monitoring. No experimental infection, treatment, or manipulation of live animals was performed for the purposes of this study. Therefore, institutional ethical approval was not required for this type of sampling. The bacterial isolates were subsequently handled according to institutional guidelines and standard microbiological safety practices. Permission to collect and use the samples for research purposes was obtained from the farm owner.

## Data Availability

NCBI-Genbank-SRAOceanimonas baumannii strain:ICA-29 Genome sequencing (Original data)

## Appendix Supplementary materials

Image, application 1

Image, application 2

Image, application 3
